# Supplementary material for: Natural grazing by horses and cattle promotes bird diversity in a restored European alluvial grassland
Source: PeerJ. 2024 Jul 19;12:e17777. doi: 10.7717/peerj.17777 (PMC11262302; doi:10.7717/peerj.17777)
Supplement: Supplemental Information 1 [file peerj-12-17777-s001.docx]

**Supplementary material**

**Natural grazing by horses and cattle promotes bird diversity in a restored European alluvial grassland**

Journal: PeerJ

Authors: Lovász, L, Korner-Nievergelt, F, Amrhein, V.

Corresponding author email: lilla.lovasz@unibas.ch; lovasz.lilla@yahoo.com

**Table 1** Criteria for classifying the bird species of our main study area into guilds

**Table 2** List of species and number of individuals counted on the Rhine island (on the main and control site)

**Suppl. Fig. 1** Bird observation transects in the main study site (yellow) and in the control sites (white). The dashed yellow line indicates the fence around the main study site, while the dotted white lines are approximate boundaries of the sampled area (as no fence existed at the time of the surveys). (Base map: Google Maps, Map data ©2024 GeoBasis-DE/BKG ©2009, Google France)

**Suppl. Fig. 2** Abundance of birds of the four guilds in relation to land cover types. Number of individuals (grey dots) and average number of individuals (regression lines, with 95% compatibility intervals represented as dotted lines): a) aerial foraging birds; b) open-area foraging birds; c) wetland-foraging birds; d) woodland-foraging birds

**Suppl. Fig. 3** Number of species and average number of species per grid cell in relation to grazer density on the median day of the study period (1^st^ June). Left figure: horizontal boxplots are the number of species per grid cell per survey. Sample sizes refer to the sum of the number of grid cells where the respective number of species was found during the surveys. On the right, the regression line is the average number of species per grid cell, and dotted lines are compatibility intervals

**Parameter estimates of the model parameters of all models used**

**(Respective figures: Fig 4 and Suppl. Fig 1a-d)**

Parameter estimates (median, lower and upper limits of the 95% CI) of the model for **species richness**, a zero-inflated negative binomial additive mixed model. All predictor variables were standardised (so that their mean was zero and their standard deviation one) except surface water that was transformed into an indicator variable. Additionally to the predictors presented in the table, a two-dimensional tensor product smooth was used for grazing intensity and date presented in **Fig 4**.

50% 2.5% 97.5%

*Bernoulli model for the zero values*

b_zi_Intercept -3.21 -6.51 -2.01

*Negative binomial model for the counts*

*Fixed effects*

b_Intercept -1.87 -2.18 -1.60

b_size.log.z 0.76 0.57 0.96

b_Cover.tree.z 0.34 0.04 0.65

b_Cover.bush.z 0.21 -0.04 0.44

b_Cover.sapling.z 0.00 -0.12 0.11

b_Bareground.z -0.21 -0.34 -0.09

b_Surface.water.bin 0.79 0.52 1.08

*Variance parameters*

sd_cellnr__Intercept 0.59 0.49 0.72

shape 13.76 4.94 146.35

Parameter estimates (median, lower and upper limits of the 95% CI) of the model for **abundance of aerial foraging birds**, a zero-inflated negative binomial additive model. All predictor variables were standardised (so that their mean was zero and their standard deviation one) except surface water that was transformed into an indicator variable. Additionally to the predictors presented in the table, a two-dimensional tensor product smooth was used for grazing intensity and date presented in **Suppl. Fig 1a**. When including the ID of the grid cell as a random factor to account for repeated measure of the same grid cells, the among-grid-cell variance was close to zero, and therefore we dropped that random term from the model.

50% 2.5% 97.5%

*Bernoulli model for the zero values*

b_zi_Intercept 0.64 -3.08 3.02

*Negative binomial model for the counts*

*Fixed effects*

b_Intercept -4.99 -7.80 -2.39

b_Cover.tree.z -0.36 -1.16 0.55

b_Cover.bush.z -0.03 -0.82 0.84

b_Cover.sapling.z -0.44 -0.81 -0.03

b_Bareground.z -0.19 -0.57 0.23

b_Surface.water.bin -0.70 -1.50 0.12

*Variance parameters*

shape 0.02 0.01 0.23

Parameter estimates (median, lower and upper limits of the 95% CI) of the model for **abundance of birds foraging on open areas**, a zero-inflated negative binomial additive model. All predictor variables were standardised (so that their mean was zero and their standard deviation one) except surface water that was transformed into an indicator variable. Additionally to the predictors presented in the table, a two-dimensional tensor product smooth was used for grazing intensity and date presented in **Suppl. Fig 1b**. When the ID of the grid cell was included as a random factor in the model, the model did not converge, most likely because the among-grid-cell variance was close to zero. To assess the amount of pseudoreplication due to repeated measures of the same grid cell, we calculated the inter-cell correlation coefficient in the model residuals, which was 0.007.

50% 2.5% 97.5%

*Bernoulli model for the zero values*

b_zi_Intercept -2.80 -6.21 -1.00

*Negative binomial model for the counts*

*Fixed effects*

b_Intercept -1.31 -1.83 -0.90

b_Cover.tree.z 0.06 -0.28 0.41

b_Cover.bush.z 0.28 0.06 0.51

b_Cover.sapling.z -0.17 -0.28 -0.06

b_Bareground.z -0.11 -0.22 0.01

b_Surface.water.bin 0.47 0.22 0.74

*Variance parameters*

shape 0.06 0.05 0.08

Parameter estimates (median, lower and upper limits of the 95% CI) of the model for **abundance of wetland-foraging birds**, a zero-inflated negative binomial additive model. All predictor variables were standardised (so that their mean was zero and their standard deviation one) except surface water that was transformed into an indicator variable. Additionally to the predictors presented in the table, a two-dimensional tensor product smooth was used for grazing intensity and date presented in **Suppl. Fig 1c**. When the ID of the grid cell was included as a random factor in the model, the model did not converge, most likely because the among-grid-cell variance was close to zero. To assess the amount of pseudoreplication due to repeated measures of the same grid cell, we calculated the inter-cell correlation coefficient in the model residuals, which was 0.08.

50% 2.5% 97.5%

*Bernoulli model for the zero values*

b_zi_Intercept -2.60 -5.89 -0.92

*Negative binomial model for the counts*

*Fixed effects*

b_Intercept -3.34 -3.81 -2.90

b_Cover.tree.z 0.60 0.28 0.92

b_Cover.bush.z 0.19 -0.05 0.44

b_Cover.sapling.z -0.52 -0.72 -0.34

b_Bareground.z -0.39 -0.52 -0.26

b_Surface.water.bin 2.29 2.02 2.56

*Variance parameters*

shape 0.11 0.09 0.15

Parameter estimates (median, lower and upper limits of the 95% CI) of the model for **abundance of woodland-foraging birds**, a zero-inflated negative binomial additive model. All predictor variables were standardised (so that their mean was zero and their standard deviation one) except surface water that was transformed into an indicator variable. Additionally to the predictors presented in the table, a two-dimensional tensor product smooth was used for grazing intensity and date presented in **Suppl. Fig 1d**. When the ID of the grid cell was included as a random factor in the model, the model did not converge, most likely because the among-grid-cell variance was close to zero. To assess the amount of pseudoreplication due to repeated measures of the same grid cell, we calculated the inter-cell correlation coefficient in the model residuals, which was 0.03.

50% 2.5% 97.5%

*Bernoulli model for the zero values*

b_zi_Intercept -2.84 -6.12 -1.19

*Negative binomial model for the counts*

*Fixed effects*

b_Intercept -1.15 -1.62 -0.78

b_Cover.tree.z 0.86 0.62 1.13

b_Cover.bush.z 0.42 0.24 0.60

b_Cover.sapling.z 0.14 0.05 0.23

b_Bareground.z -0.19 -0.29 -0.09

b_Surface.water.bin 0.10 -0.12 0.33

*Variance parameters*

shape 0.11 0.09 0.14
