# Supplementary material for: Natural grazing by horses and cattle promotes bird diversity in a restored European alluvial grassland
Source: PeerJ. 2024 Jul 19;12:e17777. doi: 10.7717/peerj.17777 (PMC11262302; doi:10.7717/peerj.17777)
Supplement: Supplemental Information 2 [file peerj-12-17777-s002.docx]

**Table 1** Criteria for classifying the bird species of our main study area into guilds

| *Foraging guild* | **Aerial** | **Open area** | **Wetland** | **Woodland** |
| --- | --- | --- | --- | --- |
| *Criteria of classification* | Species mainly capturing their prey and foraging in the air, without the need of landing | Species mainly foraging on the ground where vegetation is scarce or low | Species whose main foraging strategy is dependent on the proximity to water, including reedbeds | Species mainly foraging in areas partially or entirely covered by shrubs and/or trees |
