# Supplementary material for: Natural grazing by horses and cattle promotes bird diversity in a restored European alluvial grassland
Source: PeerJ. 2024 Jul 19;12:e17777. doi: 10.7717/peerj.17777 (PMC11262302; doi:10.7717/peerj.17777)
Supplement: Supplemental Information 3 [file peerj-12-17777-s003.docx]

**Table 2** List of species and number of individuals counted on the Rhine island (on the main and control site)

|  | *Species name (Latin)* | Species name (English) | Foraging guild | Number of individuals |
| --- | --- | --- | --- | --- |
| 1 | *Accipiter gentilis* | Northern goshawk | woodland | 1 |
| 2 | *Accipiter nisus* | Eurasian sparrowhawk | woodland | 4 |
| 3 | *Acrocephalus scirpaceus* | Eurasian reed warbler | wetland | 8 |
| 4 | *Aegithalos caudatus* | Long-tailed tit | woodland | 41 |
| 5 | *Alauda arvensis* | Eurasian skylark | open | 640 |
| 6 | *Alcedo atthis* | Common kingfisher | wetland | 33 |
| 7 | *Alopochen aegyptiaca* | Egyptian goose | wetland | 103 |
| 8 | *Anas crecca* | Eurasian teal | wetland | 34 |
| 9 | *Anas platyrhynchos* | Mallard | wetland | 172 |
| 10 | *Anser anser* | Greylag goose | wetland | 3 |
| 11 | *Anthus sp* | Pipit species | open | 475 |
| 12 | *Apus apus* | Common swift | aerial | 275 |
| 13 | *Ardea cinerea* | Grey heron | wetland | 65 |
| 14 | *Aythya fuligula* | Tufted duck | wetland | 140 |
| 15 | *Aythya nyroca* | Ferruginous Duck | wetland | 11 |
| 16 | *Branta canadensis* | Canada goose | wetland | 2 |
| 17 | *Buteo buteo* | Common buzzard | open | 6 |
| 18 | *Carduelis carduelis* | European goldfinch | woodland | 43 |
| 19 | *European greenfinch* | European greenfinch | woodland | 16 |
| 20 | *Certhia sp* | Treecreeper species | woodland | 1 |
| 21 | *Charadrius dubius* | Little ringed plover | wetland | 4 |
| 22 | *Ciconia ciconia* | White stork | open | 37 |
| 23 | *Columba palumbus* | Common wood pigeon | woodland | 2 |
| 24 | *Corvus corone* | Carrion crow | open | 29 |
| 25 | *Corvus corax* | Common raven | woodland | 7 |
| 26 | *Common cuckoo* | Cuculus canorus | woodland | 1 |
| 27 | *Cygnus olor* | Mute swan | wetland | 235 |
| 28 | *Delichon urbicum* | Common house martin | aerial | 138 |
| 29 | *Dendrocopos major* | Great spotted woodpecker | woodland | 11 |
| 30 | *Dendrocoptes medius* | Middle spotted woodpecker | woodland | 1 |
| 31 | *Dryobates minor* | Lesser spotted woodpecker | woodland | 3 |
| 32 | *Egretta alba* | Great egret | wetland | 5 |
| 33 | *Egretta garzetta* | Little egret | wetland | 7 |
| 34 | *Emberiza citrinella* | Yellowhammer | woodland | 14 |
| 35 | *Emberiza schoeniclus* | Common reed bunting | wetland | 30 |
| 36 | *Erithacus rubecula* | European robin | woodland | 113 |
| 37 | *Falco subbuteo* | Eurasian hobby | aerial | 2 |
| 38 | *Falco tinnunculus* | Common kestrel | open | 3 |
| 39 | *Ficedula hypoleuca* | European pied flycatcher | woodland | 2 |
| 40 | *Fringilla coelebs* | Common chaffinch | woodland | 185 |
| 41 | *Fulica atra* | Eurasian coot | wetland | 22 |
| 42 | *Gallinago gallinago* | Common snipe | wetland | 10 |
| 43 | *Garrulus glandarius* | Eurasian jay | woodland | 15 |
| 44 | *Hirundo rustica* | Barn swallow | aerial | 422 |
| 45 | *Jynx torquilla* | Eurasian wryneck | woodland | 3 |
| 46 | *Lanius collurio* | Red-backed shrike | woodland | 142 |
| 47 | *Lanius excubitor* | Great grey shrike | woodland | 3 |
| 48 | *Locustella luscinioides* | Savi's warbler | wetland | 1 |
| 49 | *Milvus migrans* | Black kite | open | 8 |
| 50 | *Milvus milvus* | Red kite | open | 1 |
| 51 | *Motacilla alba* | White wagtail | open | 109 |
| 52 | *Motacilla cinerea* | Grey wagtail | wetland | 17 |
| 53 | *Motacilla flava* | Western yellow wagtail | open | 4 |
| 54 | *Netta rufina* | Red-crested pochard | wetland | 6 |
| 55 | *Oenanthe oenanthe* | Northern Wheatear | open | 1 |
| 56 | *Parus caeruleus* | Eurasian blue tit | woodland | 64 |
| 57 | *Parus major* | Great tit | woodland | 285 |
| 58 | *Phasianus colchicus* | Common pheasant | open | 5 |
| 59 | *Philomachus pugnax* | Ruff | wetland | 1 |
| 60 | *Phoenicurus ochruros* | Black redstart | open | 3 |
| 61 | *Phoenicurus phoenicurus* | Common redstart | open | 1 |
| 62 | *Phylloscopus collybita* | Common chiffchaff | woodland | 34 |
| 63 | *Phylloscopus trochilus* | Willow warbler | woodland | 2 |
| 64 | *Picus viridis* | European green woodpecker | open | 1 |
| 65 | *Prunella modularis* | Dunnock | woodland | 8 |
| 66 | *Regulus ignicapilla* | Common firecrest | woodland | 2 |
| 67 | *Regulus regulus* | Goldcrest | woodland | 3 |
| 68 | *Saxicola rubetra* | Whinchat | open | 4 |
| 69 | *Spatula clypeata* | Northern shoveler | wetland | 3 |
| 70 | *Spinus spinus* | Eurasian siskin | woodland | 23 |
| 71 | *Sturnus vulgaris* | Common starling | open | 940 |
| 72 | *Sylvia atricapilla* | Eurasian blackcap | woodland | 10 |
| 73 | *Sylvia borin* | Garden warbler | woodland | 7 |
| 74 | *Sylvia communis* | Common whitethroat | woodland | 4 |
| 75 | *Sylvia curruca* | Lesser whitethroat | woodland | 2 |
| 76 | *Tachybaptus ruficollis* | Little grebe | wetland | 26 |
| 77 | *Tadorna ferruginea* | Ruddy shelduck | wetland | 6 |
| 78 | *Tringa glareola* | Wood sandpiper | wetland | 5 |
| 79 | *Tringa ochropus* | Green sandpiper | wetland | 71 |
| 80 | *Tringa totanus* | Common redshank | wetland | 5 |
| 81 | *Troglodytes troglodytes* | Eurasian wren | woodland | 28 |
| 82 | *Turdus iliacus* | Redwing | woodland | 52 |
| 83 | *Turdus merula* | Common blackbird | woodland | 96 |
| 84 | *Turdus philomelos* | Song thrush | woodland | 9 |
| 85 | *Turdus pilaris* | Fieldfare | open | 89 |
| 86 | *Upupa epops* | Eurasian hoopoe | open | 1 |
| 87 | *Vanellus vanellus* | Northern lapwing | wetland | 27 |
