# Supplementary figures and images for: Natural grazing by horses and cattle promotes bird diversity in a restored European alluvial grassland
Source: PeerJ. 2024 Jul 19;12:e17777. doi: 10.7717/peerj.17777 (PMC11262302; doi:10.7717/peerj.17777)

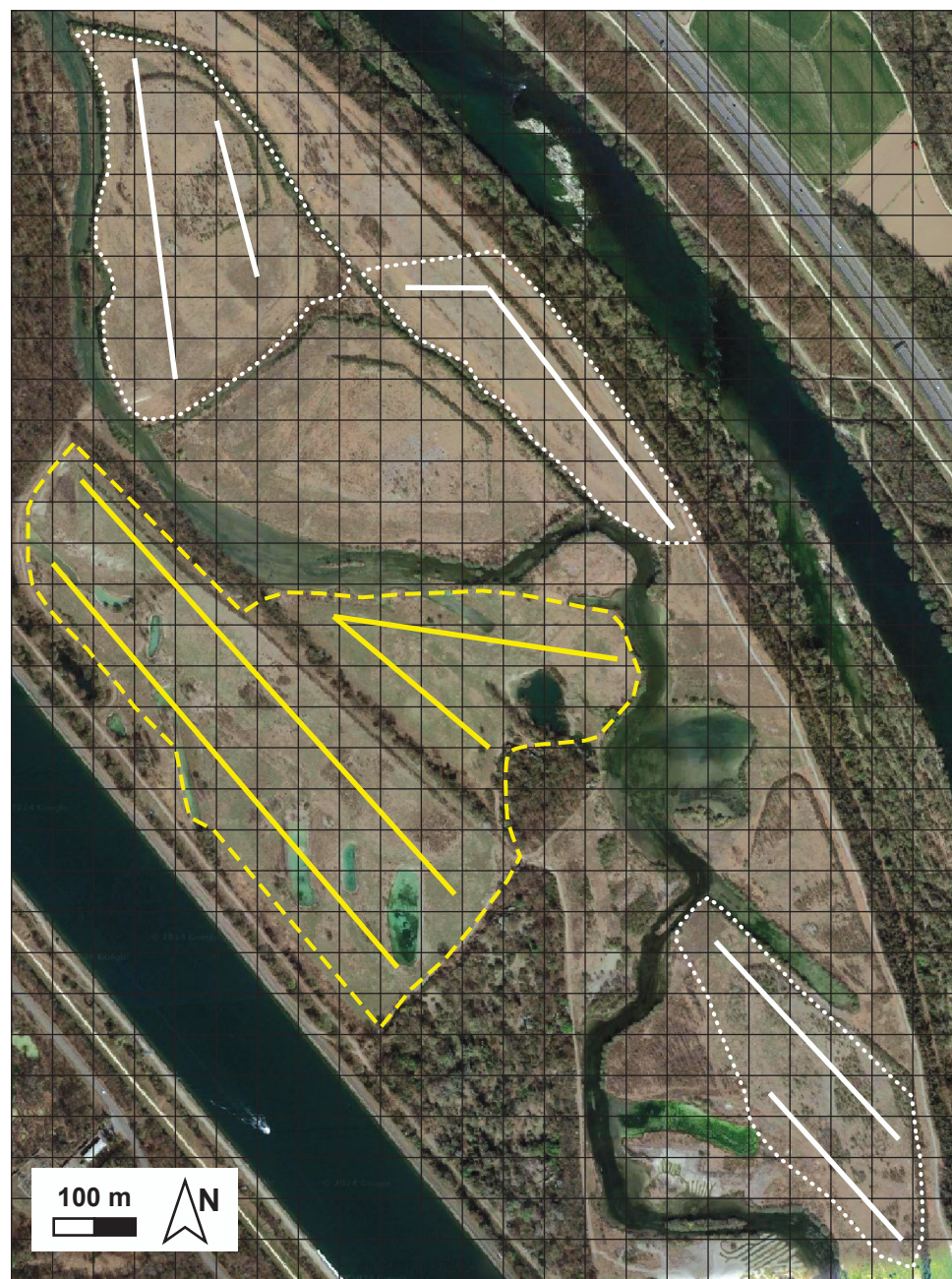

Supplement: Supplemental Information 4 — The dashed yellow line indicates the fence around the main study site, while the dotted white lines are approximate boundaries of the sampled area (as no fence existed at the time of the surveys). [file peerj-12-17777-s004.pdf]

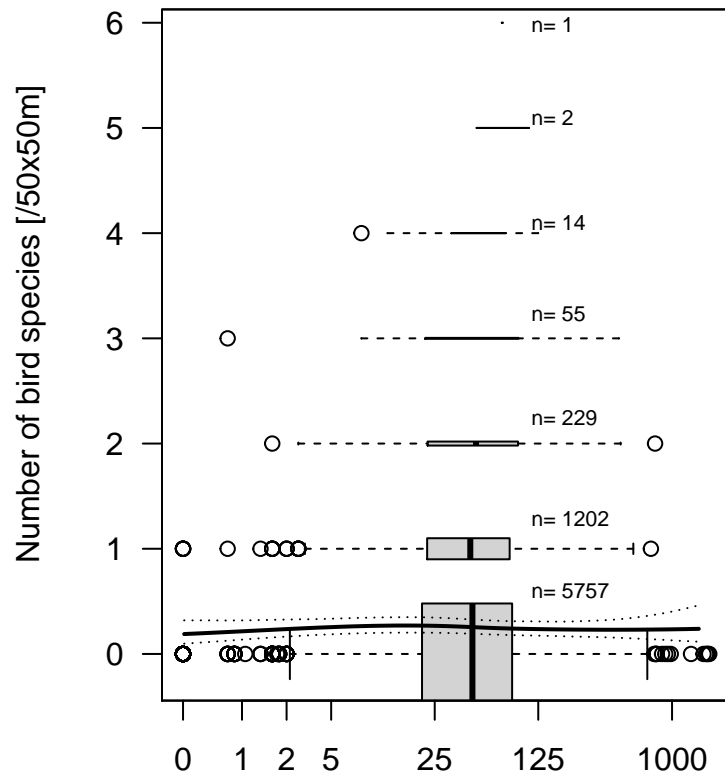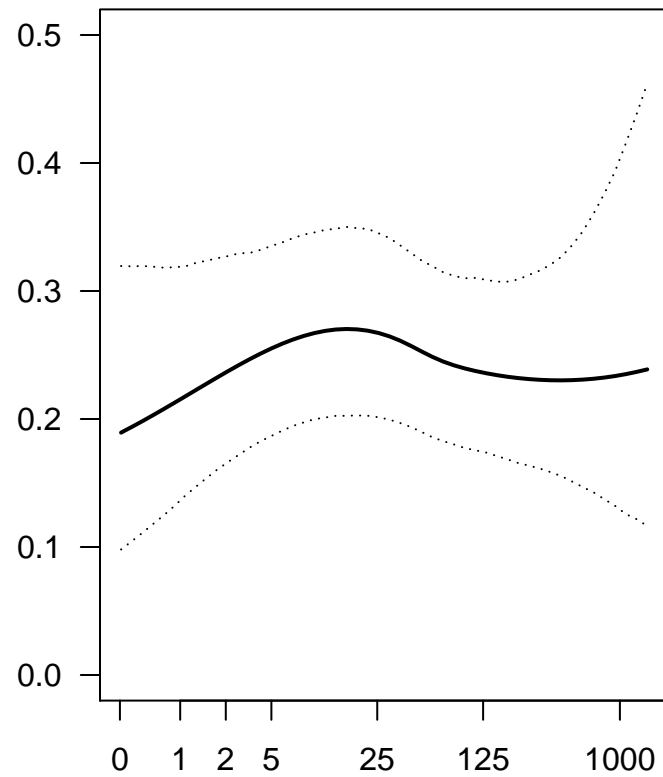

Grazer density [sum positions over 3 weeks/50x50m]

Supplement: Supplemental Information 6 — Left figure: horizontal boxplots are the number of species per grid cell per survey. Sample sizes refer to the sum of the number of grid cells where the respective number of species was found during the surveys. On the right, the regression line is the average number of species per grid cell, and dotted lines are compatibility intervals [file peerj-12-17777-s006.pdf]
