# Supplementary material for: Natural grazing by horses and cattle promotes bird diversity in a restored European alluvial grassland
Source: PeerJ. 2024 Jul 19;12:e17777. doi: 10.7717/peerj.17777 (PMC11262302; doi:10.7717/peerj.17777)

Figure 1a) Number of individuals (abundance) of the aerial-foraging birds in relation to landcover types

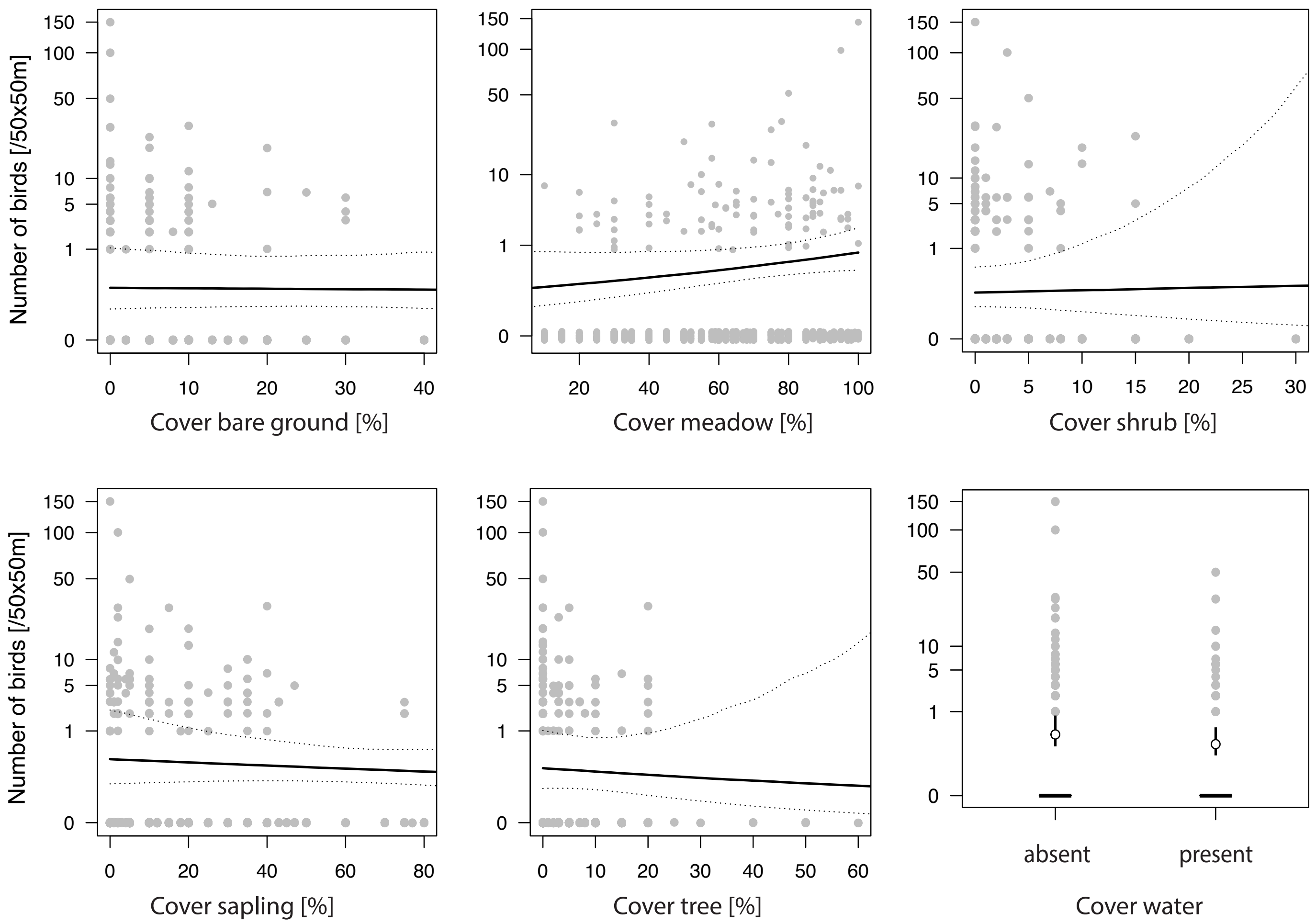

Figure 1b) Number of individuals (abundance) of birds foraging in open aeras in relation to landcover types

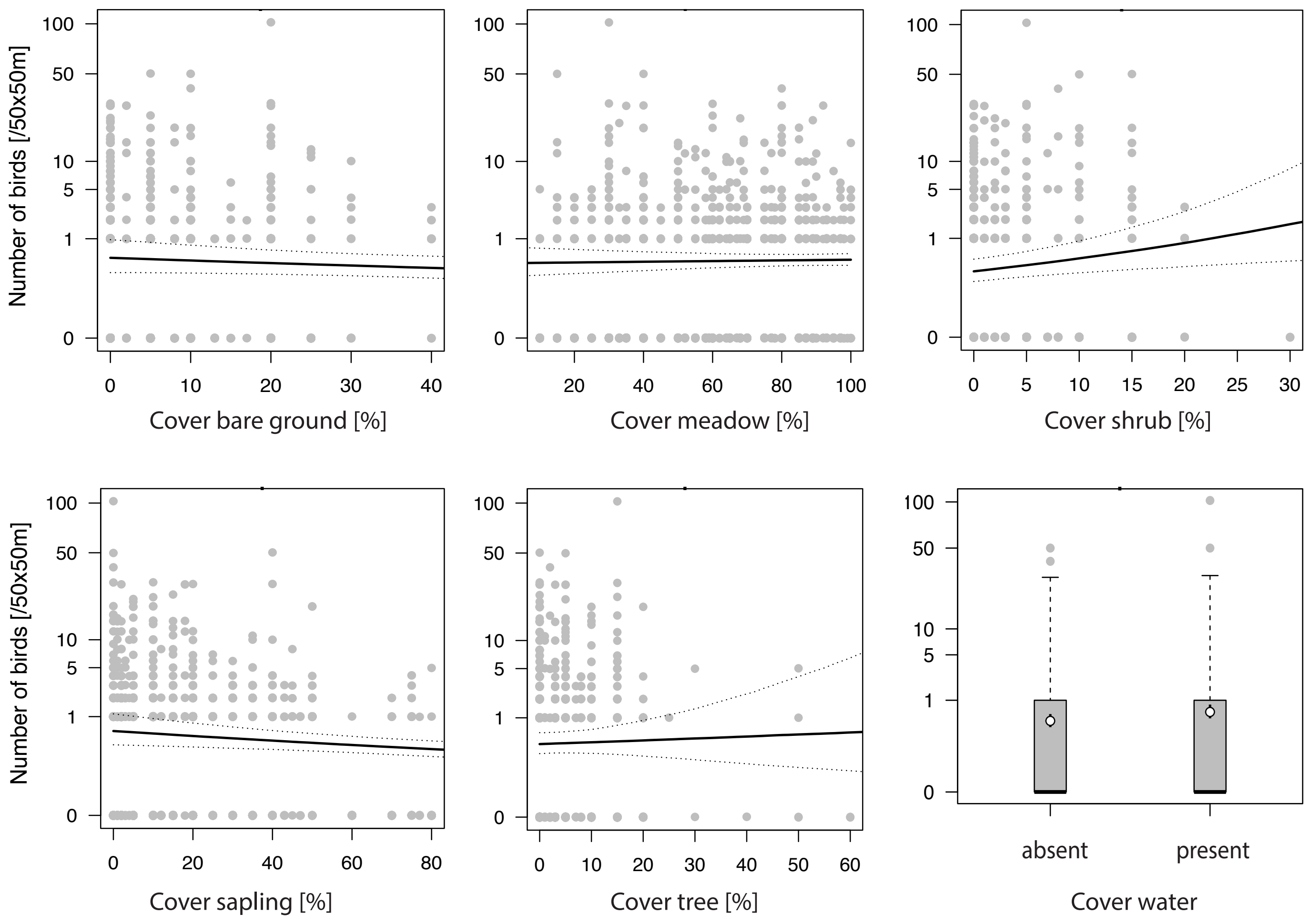

Figure 1c) Number of individuals (abundance) of the wetland-foraging birds in relation to landcover types

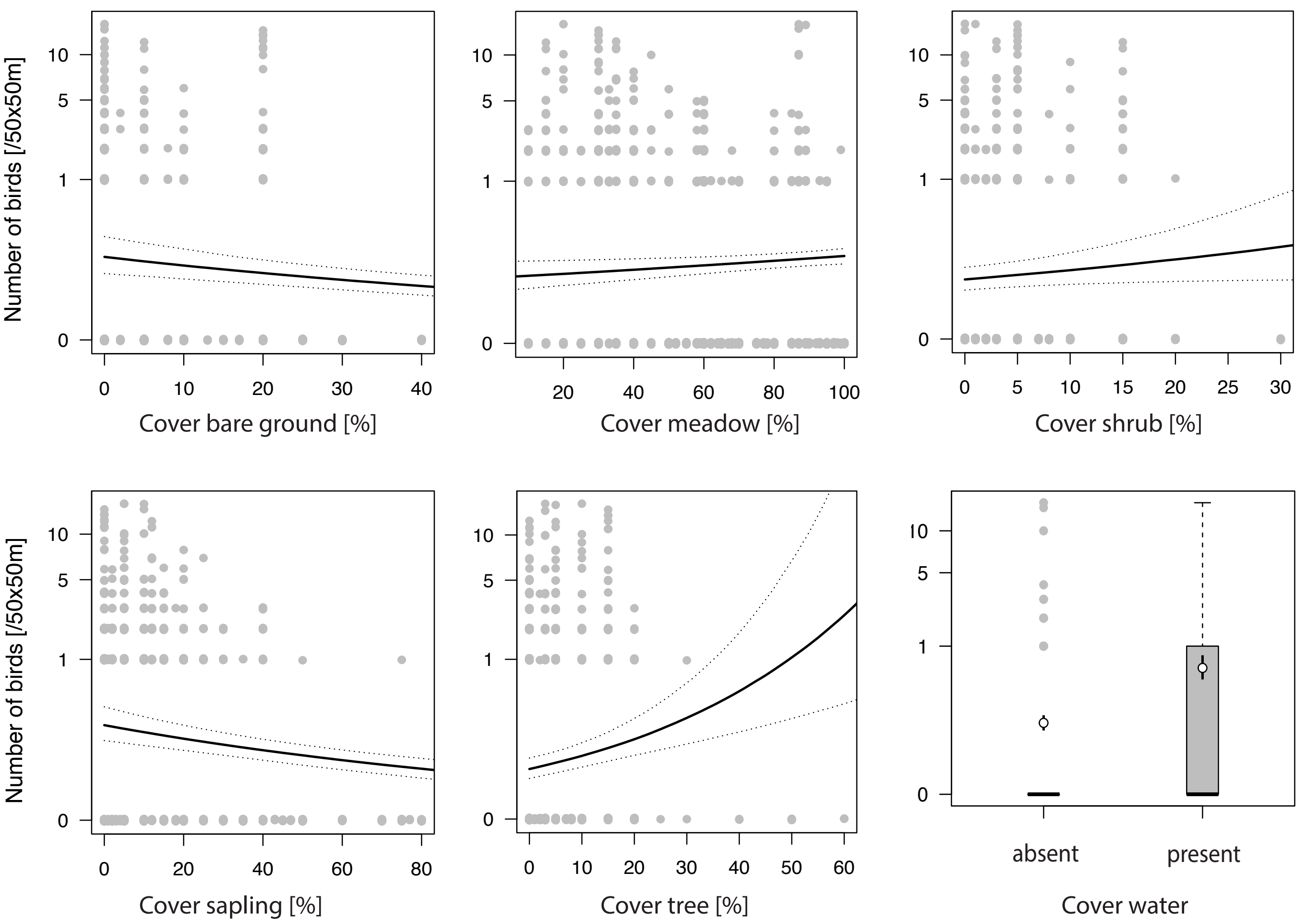

Figure 1d) Number of individuals (abundance) of the woodland-foraging birds in relation to landcover types

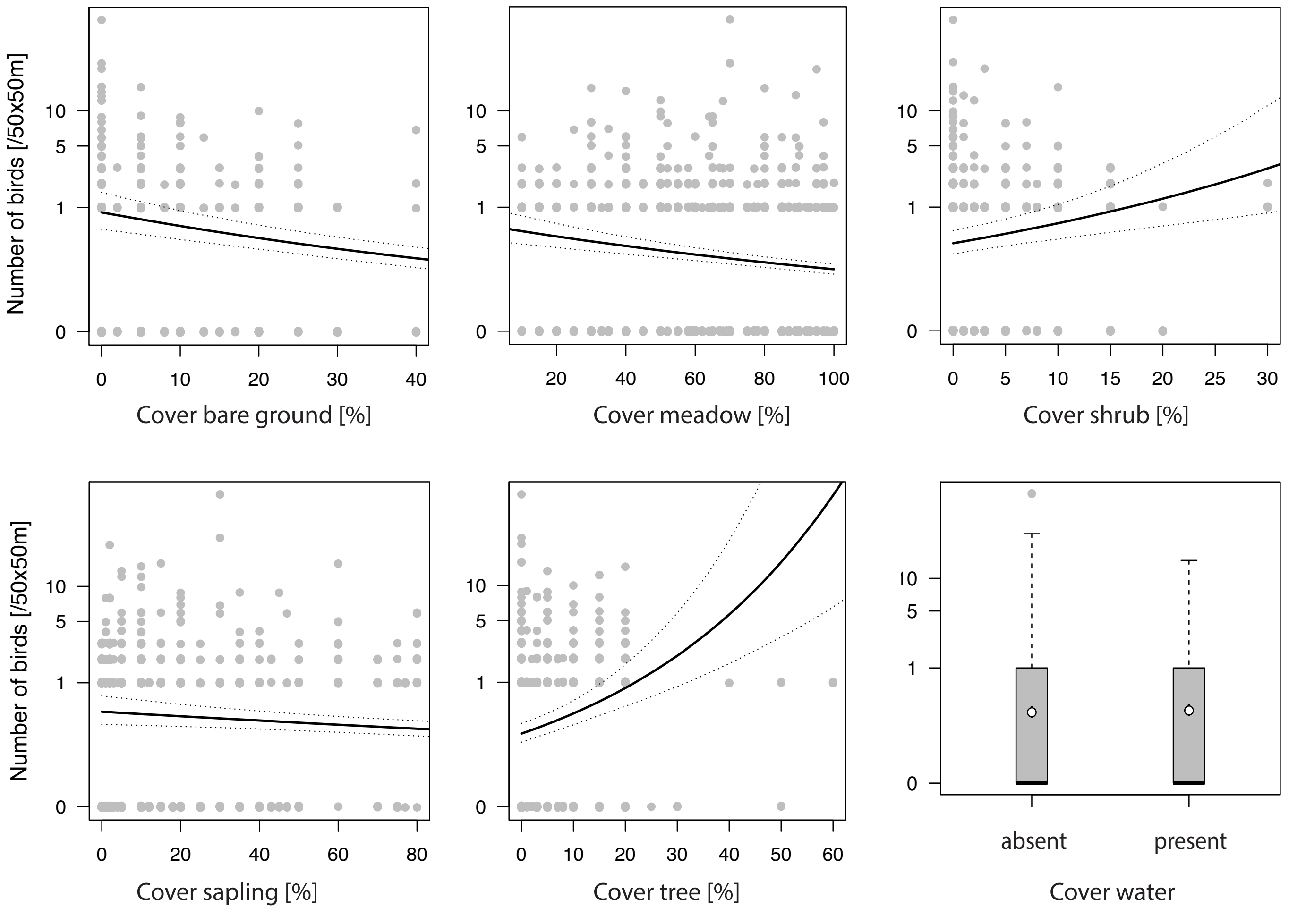

Supplement: Supplemental Information 5 — Number of individuals (grey dots) and average number of individuals (regression lines, with 95% compatibility intervals represented as dotted lines): (A) aerial foraging birds; (B) open-area foraging birds; (C) wetland-foraging birds; (D) woodland-foraging birds [file peerj-12-17777-s005.pdf]
